# How Sound Symbolism Is Processed in the Brain: A Study on Japanese Mimetic Words

**DOI:** 10.1371/journal.pone.0097905

**Published:** 2014-05-19

**Authors:** Junko Kanero, Mutsumi Imai, Jiro Okuda, Hiroyuki Okada, Tetsuya Matsuda

**Affiliations:** 1 Department of Psychology, Temple University, Philadelphia, Pennsylvania, United States of America; 2 Faculty of Environment and Information Studies, Keio University at Shonan-Fujisawa, Endo, Fujisawa, Kanagawa, Japan; 3 Department of Intelligent Systems, Faculty of Computer Science and Engineering, Kyoto Sangyo University, Kamigamo-Motoyama, Kita-Ku, Kyoto, Japan; 4 Department of Mechanical Systems, College of Engineering, Tamagawa University, Machida, Tokyo, Japan; 5 Tamagawa University Brain Science Institute, Machida, Tokyo, Japan; University of Toyama, Japan

## Abstract

Sound symbolism is the systematic and non-arbitrary link between word and meaning. Although a number of behavioral studies demonstrate that both children and adults are universally sensitive to sound symbolism in mimetic words, the neural mechanisms underlying this phenomenon have not yet been extensively investigated. The present study used functional magnetic resonance imaging to investigate how Japanese mimetic words are processed in the brain. In Experiment 1, we compared processing for motion mimetic words with that for non-sound symbolic motion verbs and adverbs. Mimetic words uniquely activated the right posterior superior temporal sulcus (STS). In Experiment 2, we further examined the generalizability of the findings from Experiment 1 by testing another domain: shape mimetics. Our results show that the right posterior STS was active when subjects processed both motion and shape mimetic words, thus suggesting that this area may be the primary structure for processing sound symbolism. Increased activity in the right posterior STS may also reflect how sound symbolic words function as both linguistic and non-linguistic iconic symbols.

## Introduction

Traditional linguistics assumes that language is independent from perceptual, motor, or affective experience and that pairings between a word’s sound and its meaning are arbitrary [1]. The notion of sound symbolism, however, challenges this well-accepted belief by suggesting natural and systematic relationships between word sound and meaning [2]. People across the world intuitively associate the nonsense word “baluma” to a round shape and “takete” to a spiky shape (i.e., bouba/kiki effect) [3]–[4]. Since then, a large body of linguistic and psychological research has investigated sound symbolism (e.g., [5]). Sound symbolic words are found in many languages including English. For example, *bump* and *thump* have sounds similar to their meanings–an event with an abrupt end [6]. Furthermore, a number of languages, including Japanese, have a large grammatically defined word class in which sound symbolism is apparent. These sound symbolic words, which are called mimetics, idiophones, or expressives, are abundant in African [7] and East Asian languages [8]–[14]. Adults [15]–[16], as well as infants and toddlers [17]–[22], are sensitive to sound symbolism in mimetic words, regardless of the language they speak. For example, the sound symbolism of Japanese mimetic words promotes verb learning in both Japanese- and English-reared children [18]–[20]. The existence of sound symbolism across languages has led some researchers to claim that this phenomenon can provide insights into the ontogenesis and phylogenesis of language [4], [18], [23]. Despite its significance, the neural mechanisms of sound symbolism are yet to be sufficiently investigated.

Ramachandran and Hubbard [4] hypothesized that sound symbolism shares the neural mechanisms underlying synesthesia. They further argue that multi-sensory integration at the temporal–parietal–occipital (TPO) junction, or more specifically the angular gyrus, is the critical region for sensing sound symbolism. In addition, they noted anecdotally that individuals with damage to the angular gyrus did not show the bouba/kiki effect. Nevertheless, these ideas are largely speculative and have never been investigated empirically.

We agree with this previous hypothesis that perceiving sound symbolism requires a unique integrative process. We hypothesize, however, that the posterior part of the superior temporal sulcus (STS) is a key area in this processing. The STS represents 2 routes for conceptual access: the left STS processes linguistic sounds, whereas the right STS processes environmental sounds [24]. The universal understanding of mimetic words suggests that these words possess some features of non-linguistic environmental sounds that do not require language system for understanding. We argue that neural processing of sound symbolic words integrates the two conceptual processes involving the bilateral STS.

A previous functional magnetic resonance imaging (fMRI) study found that auditory presentation of Japanese mimetic words for animal sounds (e.g., *ka-ka*, onomatopoeia for crow croaks) more strongly activated the right STS than the names of the animals (e.g., *karasu*, “crow” in English) [25]. Similarly, Japanese mimetic words for animal sounds more strongly activated the STS bilaterally than the actual animal sounds (e.g., sound of a crow croaking). That study concluded that onomatopoeic words activate both the left and right STS because they have acoustic properties similar to real animal sounds. The acoustic similarity between mimetic words and the actual sound, however, cannot fully explain the phenomenon of sound symbolism, because sound symbolic words are not limited to mere mimicry of environmental sounds.

For example, Japanese mimetic words are roughly classified into 3 categories–phonomimes, phenomimes, and psychomimes [26]. Phonomimes, or *giongo*, are onomatopoeia that acoustically imitate actual sound (e.g., *wanwan* for dog barking). Phenomimes, or *gitaigo*, represent the characteristics of input from non-auditory senses (e.g., *yotayota* for walking clumsily). Psychomimes, or *gijogo*, represent psychological states (e.g., *wakuwaku* for the feeling of excitement). Several studies demonstrated that Japanese as well as non-Japanese speakers can discern sound-meaning correspondences in the latter two types of mimetics [18]–[20], [27], [28]. Sound symbolism in English, such as *squeeze*, *squirt*, *squint*, *bump*, *thump*, and *plump* [6], are found beyond the non-auditory domain as well. Thus, in order to fully understand the neural processing of sound symbolism, we must investigate sound symbolism in the non-auditory domain.

We hypothesize that right STS participation can differentiate sound symbolic words from non-sound symbolic words. Therefore, all types of mimetic words, including phenomimes and psychomimes, should activate the right STS. To determine whether the right STS is the primary structure for sound symbolism processing, we investigated whether this region responds to non-onomatopoeic mimetic words. For this purpose, we tested mimetics in two domains, motion and shape, and all words were presented visually rather than auditorily. Experiment 1 contrasted Japanese mimetic words with non-sound symbolic conventional verbs and adverbs, all of which express aspects of human motion. Experiment 2 compared the neural processing of mimetic words for human motion as well as for shape to ensure that the right STS activation is not limited to the domain of motion. Interpretation of the STS activation in Experiment 1 requires caution because the STS shows activation during the processing of animated figures [29], [30] and point-light biological motion [31]. If the right STS is the key structure for sound symbolism processing, we should see the activation of this area both for motion mimetic words and for shape mimetic words. Experiment 2 tested mimetic words only, as differences in brain activation across word classes (mimetic words, verbs, and adverbs) were demonstrated in Experiment 1, and as with inclusion of multiple word classes would substantially increase the length of each scanning session.

## Materials and Methods

### Experiment 1

#### Participants

Sixteen native Japanese speakers aged 22–25 years (7 women, 9 men; mean age = 23.7 years) participated in this study. All participants were right-handed, had normal or corrected-to-normal vision, and had no history of neurological or psychiatric symptoms. Data from 5 participants were excluded due to artifact (e.g., head movements >3 mm) or inadequate task performance (e.g., failing to press buttons as instructed during scanning sessions); thus data were analyzed from the remaining 11 participants (4 women, 7 men; age range = 22–25 years; mean age = 23.4 years). The individual in this manuscript has given written informed consent to publish these case details. The study was approved by the ethics committee of Tamagawa University.

#### Design and procedure

**Figure 1:**
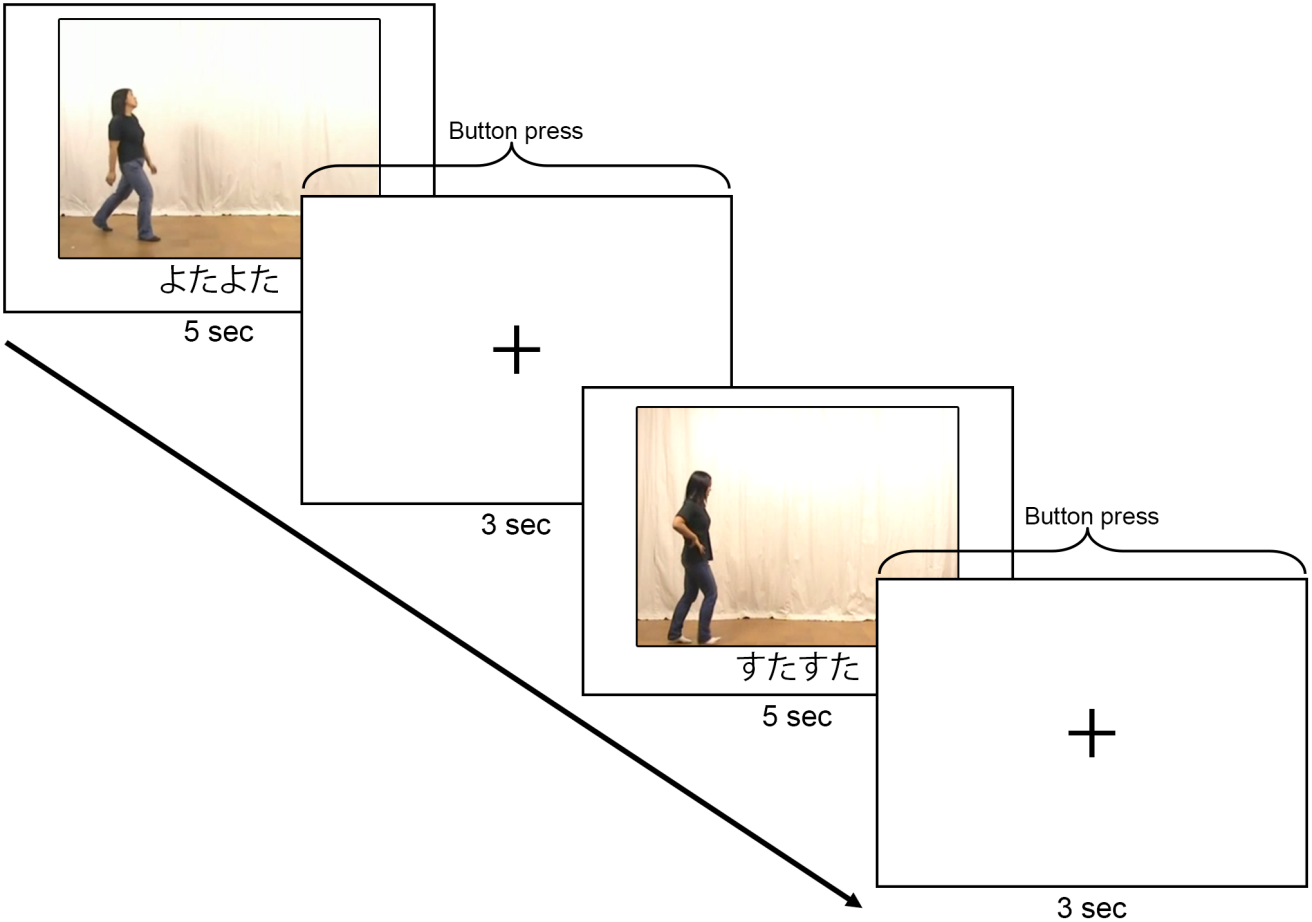
Study paradigm for Experiment 1. Experiment 1 used a blocked design. A 5-sec video clip presented a person moving from left to right and a matched/mismatched word that were followed by a 3-sec presentation of a fixation point. Participants were asked to press a button during the fixation point presentation to indicate the degree of match, on a scale of 1–5, between the motion and the mimetic words. This example shows two trials in the mimetic word block. The mimetic words depicted in this example: 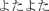 (*yotayota*) “walk clumsily” and 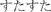 (*sutasuta*) “walk very quickly”.

Stimuli were 16 video clips of a human agent moving from left to right in different manners. Each video clip was 5-sec long, and was presented simultaneously with a sound symbolic mimetic word, a non-sound symbolic adverb, or a non-sound symbolic verb. All words were presented at the bottom of the video in *hiragana* (a type of Japanese orthographical coding in which each character represents a syllable). In half of the trials, the word and manner of motion semantically matched, whereas in the other half, the items were mismatched (e.g., the verb *aruiteiru* “to walk in the progressive aspect,” was shown with a video clip of an agent skipping). Thus, for each word class (mimetic words, verbs, or adverbs), 8 motion-word pairs were matched and 8 pairs were mismatched. Participants were instructed to determine the degree of match between the word and the motion as the video clips were presented. After each video clip, a fixation point appeared on the screen for 3 sec, and participants indicated the degree of match between the word and the motion on a scale of 1 to 5 by pressing the appropriate button with a right-hand finger (Figure 1). As Experiment 1 used a 1.5 scanner, we used a block design to maximize sensitivity to the brain response: 4 blocks were presented for each word class (mimetic words, verbs, or adverbs), with each block consisting of 4 motion-word pairs from the same word class. The order of the blocks was rotated among participants. A fixation point was inserted for 10 sec at the end of each block.

#### Stimuli and stimulus validation

Three pretests examined 120 preselected words to ensure that the mimetic words, verbs, and adverbs were balanced in terms of imageability, familiarity, and age of acquisition (AOA). Twenty-eight participants who were native-Japanese speakers rated how imageable each word was on a scale of 1 to 7. Twenty-seven participants categorized word familiarity on a scale from 1 to 7. Twenty-two participants were asked to indicate the approximate age at which they learned words from the following 8 categories: infancy, preschool, first to third grade, fourth to sixth grade, junior high school, high school, university or college, or do not know the meaning. The pretest results indicated significant differences among the 3 word classes with respect to imageability (mimetic words: 5.28; verbs: 6.40; adverbs 5.62; *F*(2,81) = 3.11, *p*<0.05) and familiarity (mimetic words: 5.42; verbs: 6.51; adverbs: 6.08; *F*(2,78) = 3.11, *p*<0.05); although mimetic words and adverbs did not significantly differ in imageability (*t*(27) = 1.200, *p* = 0.241). The results of the AOA survey indicated that participants acquired mimetic words and verbs earlier than adverbs (mean rating scores were 1.52 for mimetics, 1.55 for verbs, and 2.93 for adverbs); however, no significant difference was found between AOA of the mimetic words and verbs (Freedman test, *p* = 0.76).

#### Materials and imaging parameters

Imaging was performed using a 1.5-T MRI scanner (SIEMENS MAGNETOM SONATA, Erlangen, Germany). A high-resolution (1×1×1 mm) T1-weighted anatomical reference image was acquired from each participant using a rapid acquisition gradient echo (MP-RAGE) sequence. Multi-slice gradient echo planar imaging (EPI) was used with a TE of 50 ms and a TR of 2000 ms. Slice-acquisition was ascending within the TR interval. The matrix acquired was 64×64 voxels with a field of view of 192 mm, resulting in an in-plane resolution of 3 mm. Slice thickness was 3 mm (20 slices, whole brain coverage).

#### fMRI data analyses

fMRI data were analyzed using SPM8 software (Wellcome Department of Imaging Neuroscience, Institute of Neurology, London, UK). The gradient-echo echo-planar images for each time series were realigned with reference to the first image acquired in each session to correct for head motion. The anatomical images were co-registered with the mean functional images and normalized to the Montreal Neurological Institute (MNI) brain template. Functional data were normalized using the same transformation parameters and smoothed in the spatial domain (isotropic Gaussian kernel of 8 mm full width half-maximum). Low-frequency drifts were removed using a high-pass filter [32], and a first order autoregressive model (AR1) [33] was applied for eliminating the temporal autocorrelation of the fMRI time series data.

The fMRI time series for each participant were analyzed using a block design approach with a general linear model. The images were sorted by trial type (matched and mismatched trials), and regions unique to mimetic processing were calculated by subtracting verbs and adverbs from mimetic words. The vectors indicating the onset and duration of each of the 3 word classes (mimetic words, verbs, and adverbs) were convolved with a hemodynamic response function. The results for the single subject analyses were then used for group analyses. Images representing the estimated cerebral effects from the [mimetic words – verbs − adverbs] for each subject were analyzed using a one-way ANOVA to determine the consistency of the effects across subjects. To ensure that the activation patterns of mismatched motion-word pairs were different, the same procedure was conducted for mismatched pairs.

### Experiment 2

#### Participants

Fifteen native Japanese speakers aged 17–26 years (8 women, 7 men; mean age: 20.93 years) participated in the fMRI study. All participants were recruited on the basis of the same criteria as in Experiment 1. Four participants were excluded from the analysis as not enough data were collected for these subjects (less than 10 trials in one condition). The final data set consisted of 5 women and 6 men (mean age: 21.13 years; range = 17–27 years).

#### Stimuli

**Figure 2:**
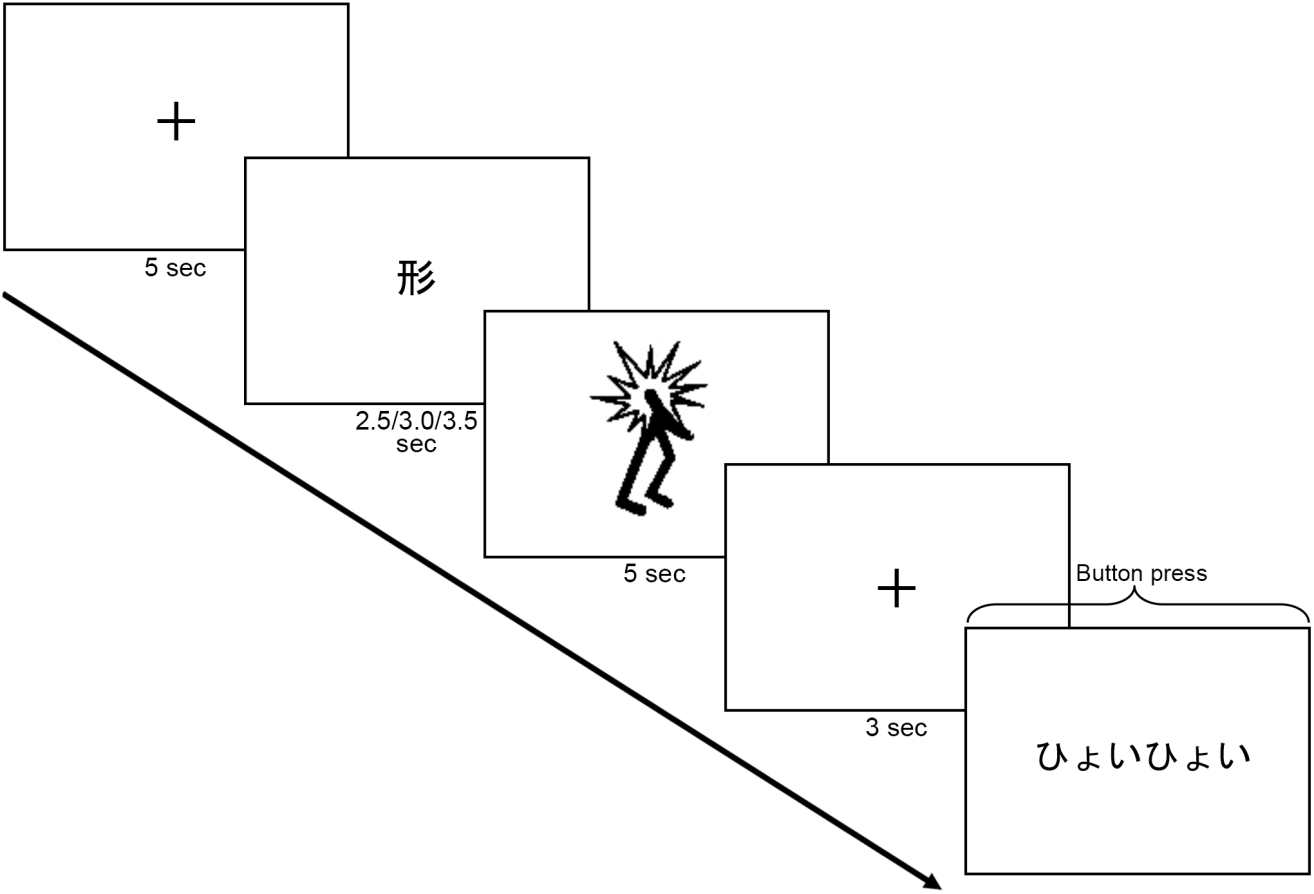
Study paradigm for Experiment 2. Experiment 2 used an event-related design. Stimuli were presented in the following order: 5-sec fixation point, 1-word instruction (presented either for 2.5, 3.0, or 3.5 sec) indicating the trial type ( 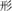 “shape” or 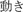 “motion”), 5-sec video clip, 3-sec fixation point, and a mimetic word. Video clips depicted an agent who stayed still in the shape trials and moved from left to right in the motion trials. During the presentation of a mimetic word, participants pressed a button to indicate the degree of match between the referent and the mimetic word. The mimetic word depicted in this example is 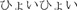 (*hyoihyoi*) which means “jumping effortlessly” in this context.

One hundred and fourteen animation clips and their corresponding mimetic words were used in the main fMRI experiment. Each video clip depicted a simple line-drawing figure with hands and legs, and this “agent” either stayed still in the center of the screen or moved from left to right on a white background (Figure 2). The still and moving images were used for the shape and motion trials, respectively.

Twenty-four mimetic words referring to human motion and 35 mimetic words referring to shape were selected from a dictionary of Japanese mimetic words (*Giongo • Gitaigo 4500 Nihongo Onomatope Jiten*) [34]. Two separate rating tests, a web-rating test and a behavioral rating test, were conducted prior to the fMRI scanning to assure that the set of experimental stimuli contained both matched motion/shape-word pairs and mismatched motion/shape-word pairs. All participants who took part in the rating tests were native Japanese speakers who did not participate in Experiments 1 or 2.

In the web-rating test, 108 participants rated the degree of match between mimetic words and shapes/manners of motion on a scale of 1 to 5. 57 participants rated the degree of match between mimetic words and manners of motion, whereas 51 participants rated the degree of match between mimetic words and shapes. Each participant was presented with 105 pairs of words and their referents. From this analysis, 50 manners of motion and 48 shape figures were selected.

After the web rating test, the remaining manners of motion and shapes were combined to create animation clips of an agent that was either motionless or that moved across the display, as described above.

In the behavioral rating test, 29 participants rated each stimuli pair (motion/shape and word) in the same manner as the scanning experiment. Thirteen shape/motion-word pairs that were judged as neither matched nor mismatched were excluded at this point. The final set of stimuli consisted of highly matched (mean rating score: 4.16 and 4.30 for the motion trials and shape trials respectively) and mismatched pairs (mean rating score: 1.34 and 1.29 for the motion trials and shape trials respectively). A total of 114 video clips (57 for each modality) were used in the fMRI experiment.

#### Design and procedure

Each shape or manner of motion appeared 1–8 times, and each shape-motion combination was different. Thus, participants saw each video clip once. A fixation point was presented for 5 sec, which was followed by a one-word instruction (either “motion” or “shape”) that directed participants to attend to either the motion or shape of the agent in the animation clip. The duration of the instruction was jittered and was 2.5, 3, or 3.5 sec; the duration for all video clips was 5 sec. After each video clip, a sound symbolic mimetic word was visually presented. In some trials, the mimetic word and indicated visual property (motion or shape) were semantically matched, but these were mismatched in other trials (e.g., a hopping motion followed by the word *yotayota* “to walk clumsily”). Participants judged the degree of match between the manner of motion in motion trials and the shape of the agent and mimetic word in shape trials. Participants pressed 1 of 5 buttons while the mimetic word was on screen. 11 Stimuli sequences were presented in pseudo-random order to control the order effects, and all words were shown in *hiragana*.

#### Imaging parameters and analysis

Scanning was performed with a 3.0-T MRI scanner (Siemens MAGNETOM Torio-Tim, Erlangen, Germany). A high-resolution (1×1×1 mm) T1-weighted anatomical reference image was acquired from each participant using a rapid acquisition gradient echo (MP-RAGE) sequence. Multi-slice gradient echo planar imaging (EPI) was used with a TE of 25 ms and a TR of 2500 ms. Slice-acquisition was ascending within the TR interval. The matrix acquired was 64×64 voxels with a field of view of 192 mm, resulting in an in-plane resolution of 3 mm. Slice thickness was 3 mm (42 slices, whole brain coverage). The acquisition window was tilted at an angle of 30° relative to the AC-PC line in order to minimize susceptibility artifacts in the orbitofrontal cortex. The fMRI data were analyzed using SPM8 software and preprocessed using the steps described for Experiment 1.

We classified the trials as matched trials with high rating scores (4 or 5) or mismatched trials with low rating scores (1 or 2). Statistical analysis of the behavioral data was performed using 2 factors: Modality (motion/shape) and Degree of Match (matched/mismatched). Thus, the trials were divided into 4 cell means: Shape-High (shape trials with a high rating score), Shape-Low (shape trials with a low rating score), Motion-High (motion trials with a high rating score), and Motion-Low (motion trials with a low rating score). For fMRI analysis, we focused on highly matched word-referent pairs, and thus highly matched trials (Shape-High and Motion-High) and mismatched trials (Shape-Low and Motion-Low) were analyzed separately. General Linear Model EPI time series were analyzed using the general linear model function implemented in SPM8. At the first level (i.e., within subjects), Shape-High, Shape-Low, Motion-High, and Motion-Low were modeled separately, creating 4 regressors. At the second level (i.e., across subjects), a one-sample *t*-test was performed on each regressor to examine the activation level.

## Results

### Experiment 1

#### Behavioral results

We examined whether the rated degree of match between the word and motion itself were comparable across the 3 word classes. For each word class, the degree of match was high for the highly matched pairs (mimetic words: 4.24; adverb: 4.30; verb: 4.09) and low for the mismatched pairs (mimetic words: 1.65; adverb: 1.64; verb: 1.10). According to a 3×2 (Word class: mimetic words/verb/adverb × matched/mismatched) ANOVA, rating scores were significantly different between the matched and mismatched pairs (*F*(1,10) = 166.06, *p*<0.01). The main effect of the word class was also significant (*F*(1,10) = 5.53, *p*<0.05). As indicated by the post-hoc test, the significant main effect for the word class was due to the difference between adverbs and verbs (*p* = 0.05, Bonferroni corrected), but the rating scores were similar between mimetic words and adverbs and between mimetic words and verbs (*p*>0.05). Reaction times (RTs) were not analyzed in Experiment 1 as participants were instructed to delay their response until each video clip was over.

#### Activation pattern for mimetic words

**Figure 3:**
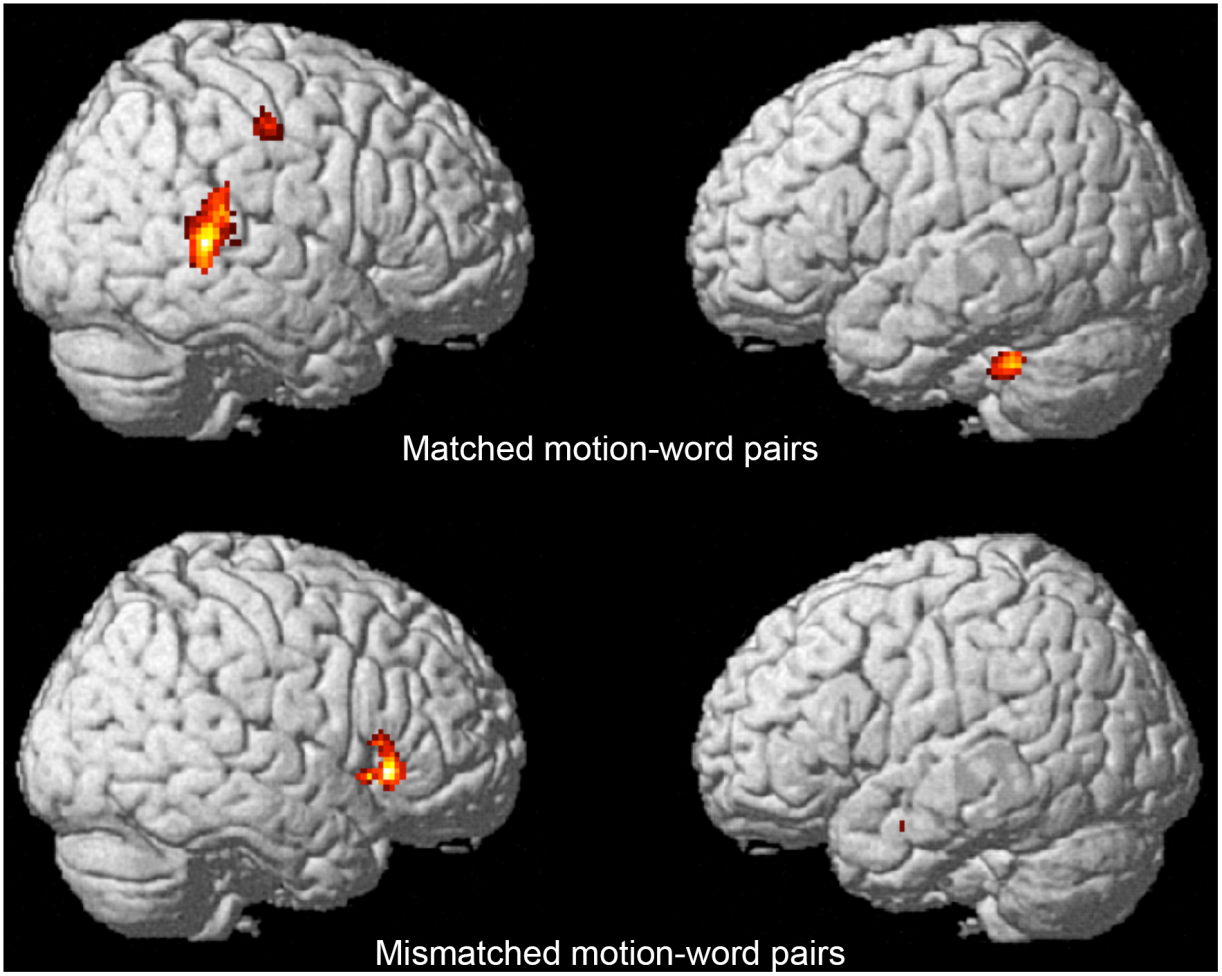
Results of Experiment 1. Regions that showed greater activation for mimetic words than for non-mimetic verbs and adverbs (*p*<0.05, FWE corrected; see Materials and Methods).

To identify the areas of activation for mimetic words, the images for verbs and adverbs were subtracted from the image of mimetic words (Figure 3; Table 1). As predicted, activation of the posterior part of the right STS was specific to mimetic words (x = 52, y = −36, z = 14; *T* = 6.53; *p*<0.05, FWE corrected). The post-central gyrus, parahippocampal gyrus, and cerebellum were also activated by mimetic words. In contrast, we confirmed the right posterior STS was not significantly activated when motion and mimetic words were mismatched (*p*>0.05, FWE corrected). Mismatched mimetic motion-word pairs showed increased activation in the parahippocampal gyrus and inferior temporal gyrus.

**Table 1:** Activation for mimetic words (Experiment 1).

Region of activation	Lat.	Coordinates			T-score	*k*
		*x*	*y*	*z*		
(Matched motion-word pairs)						
superior temporal sulcus	R	52	−36	14	6.53	398
post-central gyrus	R	40	−20	44	9.63	145
parahippocampal gyrus	L	−30	−10	−14	7.19	170
Cerebellum	L	−28	−38	−36	7.18	151
(Mismatched motion-word pairs)						
parahippocampal gyrus	L	−26	−18	−18	5.98	251
inferior frontal gyrus	R	42	22	8	5.56	288

Note: coordinates (mm) are in MNI space. L = left hemisphere; R = right hemisphere. P<0.001 (uncorrected),* k*>140,

### Experiment 2

#### Behavioral results

We examined whether Modality (motion or shape) and Degree of Match (high or low) affected RTs. Using a two-way repeated measures ANOVA, we found that the RTs were significantly longer for the motion-word pairs than for the shape-word pairs (*F*(1,10) = 7.33, *p* = 0.02). However, there was no effect of Degree of Match (*F*(1,10) = 2.13, *p* = 0.18), or interaction between Modality and Degree of Match (*F*(1,10) = 0.88, *p* = 0.37).

#### General neural activation

**Figure 4:**
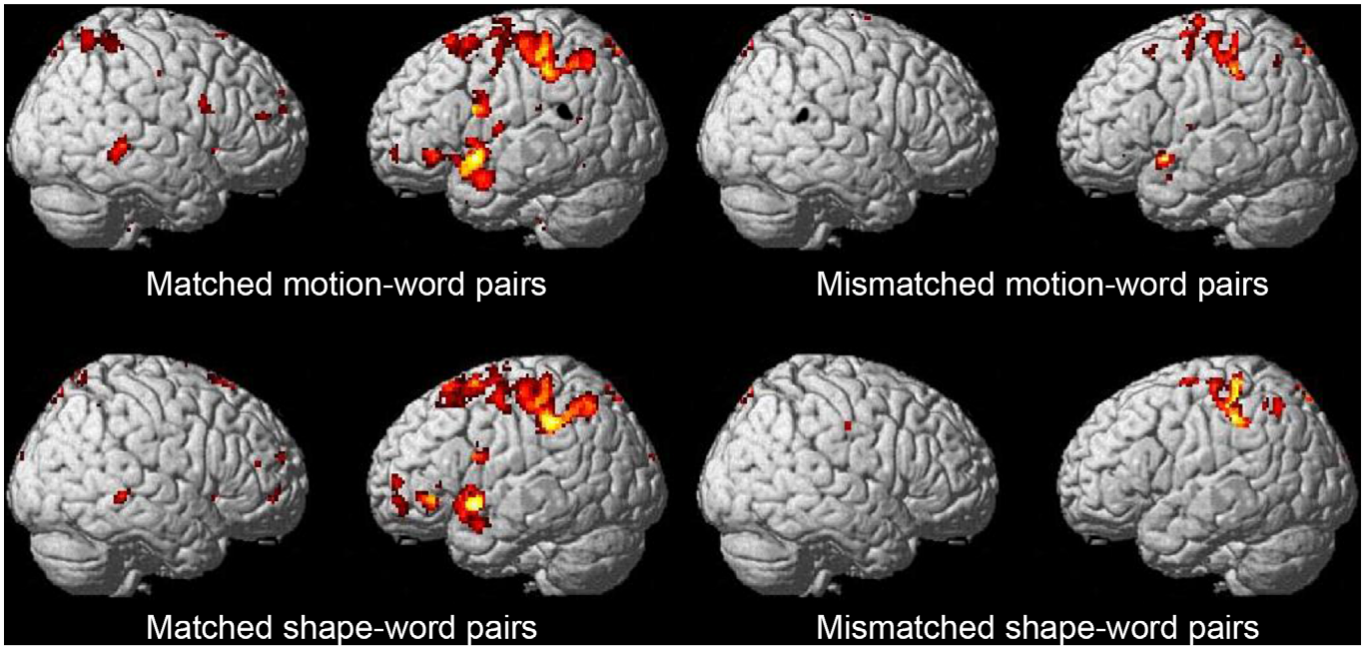
Results of Experiment 2. Several brain regions, including the right posterior STS, showed significant activation compared to baseline (*p*<0.01, FWE corrected). A strict threshold was used to ensure that the right pSTS was involved in the processing of both motion mimetic words and shape mimetic words. Brain activity observed during the motion and shape trials were not significantly different. However, the high-match trials elicited greater activation across the cortex than did low-match trials for either modality.

Several brain regions, including the right posterior STS, showed significant activation compared to baseline (Table S1; Figure 4). Importantly, brain activation observed in the motion and shape trials did not significantly differ. However, the high-match trials elicited greater activation across the cortex than did low-match trials for either modality.

#### Region of interest analysis of the right posterior STS

**Figure 5:**
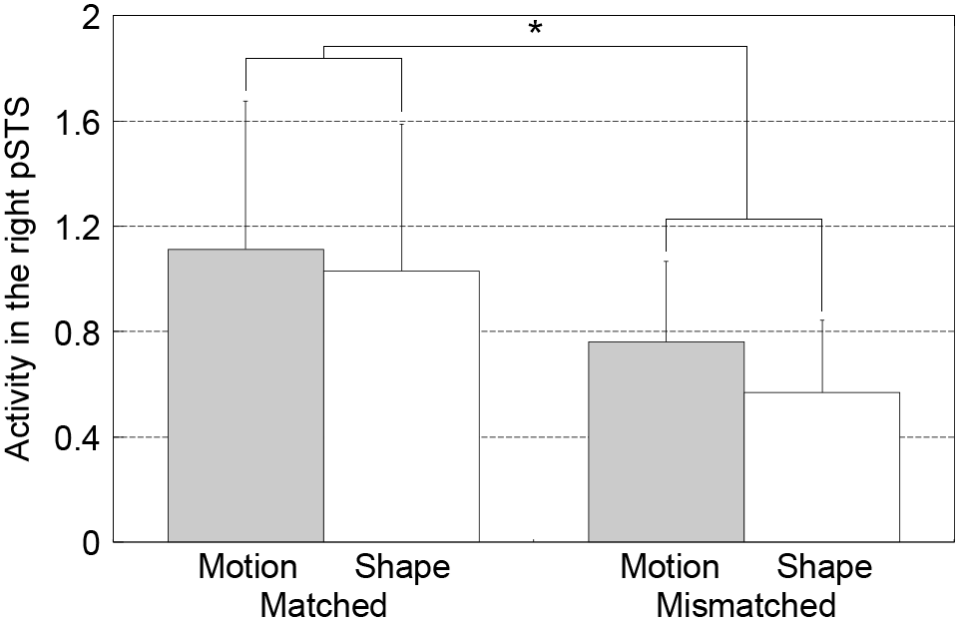
Mean beta values calculated in the ROI analysis for the right posterior STS (Experiment 2). Increased activation was observed for matched motion/shape-word pairs. A two-way ANOVA revealed the main effect of Degree of Match was statistically significant (*F*(1,10) = 8.06, *p* = 0.02). Error bars indicate ±1 standard deviation.

The right posterior STS was activated in both motion and shape trials. To investigate whether the right posterior STS was activated for all conditions (matched motion, mismatched motion, matched shape, and mismatched shape), we performed a region of interest (ROI) analysis with a 3-mm ROI located at x = 62, y  =  −38, z  =  −2 (Figure 5). This region was chosen based on the local maximum coordinates for the right posterior STS region. Although the right posterior STS was activated for all conditions compared to baseline, stronger activation was observed for matched motion/shape-word pairs. The main effect of Degree of Match was statistically significant (two-way ANOVA; *F*(1,10)  = 8.06, *p* = 0.02); however, there was no significant effect of Modality (*F*(1,10)  = 1.61, *p* = 0.23) or interaction between Modality and Degree of Match (*F*(1,10)  = 0.84, *p* = 0.38). The RTs for the Degree of Match judgment were not different across matched and mismatched pairs. The behavioral results suggest that the task difficulty did not differ between the matched and mismatched pairs in which we found a difference in neural activation.

## Discussion

In the present study, we investigated neural processing of sound symbolism using Japanese phenomimes. Despite accumulating evidence of universal sensitivity to sound symbolic word–meaning correspondences, the neural mechanism underlying this phenomenon has not been determined. Based on the idea of a functional dissociation of left and right STS [24], we hypothesized that the right posterior STS plays a critical role in sound symbolism processing. Experiment 1 tested this hypothesis by comparing Japanese mimetic words with verbs and adverbs for human motions. Supporting our hypothesis, mimetic words activated the right posterior STS even though all mimetic words were visually-presented phenomimes. This finding suggests that the function of the right posterior STS is not limited to the processing of onomatopoeia; rather, this area is likely responsible for the processing of mimetic words.

The results of Experiment 1, however, might indicate an alternative interpretation. STS is known to have multiple functions, one of which is processing biological motion. Right posterior STS activation may reflect an enhanced response to human motion caused by the paired presentation of biological motion and a mimetic word. Experiment 2 ruled out this alternative as the right posterior STS was activated not only for motion mimetic words, but also for mimetic words representing static shapes. For both motion and shape trials, the level of right posterior STS activation was higher when the mimetic word and referent were highly matched, further confirming that this region is sensitive to sound symbolism. We thus conclude that the posterior STS serves as a critical hub for processing Japanese mimetic words, and possibly sound symbolism in general.

Previous neuroimaging research on the neural processing of Japanese mimetic words focused only on the acoustic similarity between onomatopoeia (phonomimes) and environmental sounds [25] or the “embodied” explanation of sound symbolism [35]. The embodied explanation of sound symbolism suggests that a mimetic word activates a perceptual or sensorimotor area relevant to the word meaning. Similar claims have also been made for non-mimetic conventional words [36]–[43]. Thus, the embodied explanation does not explain why people sense the meaning in the sound of the word. In contrast, we suggest that sound symbolism processing requires a unique neural basis involving the posterior STS.

Although we must be cautious about drawing a reverse inference, the unique involvement of the right posterior STS in mimetic processing supports the idea that sound symbolic words are processed as both linguistic symbols and non-linguistic iconic symbols. We speculate that the posterior STS works as a hub of multimodal integration. Our view, therefore, corroborates that of Ramachandran and Hubbard [4] that linked the neural mechanisms of sound symbolism to synesthesia. Event-related potential (ERP) studies also suggest that sensory integration at the parietal-occipital regions is related to sound symbolism processing [42].

Importantly, however, Ramachandran and Hubbard [4] identified the (left) angular gyrus as the key region for high-level synesthesia. The angular gyrus and the STS are closely located but are two distinct structures. The disparity between these two claims may suggest that the distinct neural substrates are involved in sound symbolism and synesthesia. Alternatively, the nature of stimuli types may have affected the results, as sound symbolic words differ greatly in terms of modalities and level of iconicity. Our stimuli consisted of sound symbolic words that are part of the Japanese lexicon rather than nonsense sound symbolic words (e.g., “baluma” and “takete”). Conventional words and nonsense sound symbolic words may in part recruit different processing mechanisms.

Future research is required to investigate other types of sound symbolic words including psychomimes, non-mimetic words that carry sound symbolism (e.g., English sound emission verbs), and non-lexicalized sound symbolic words (e.g., “baluma” and “takete”). Comparative imaging research that includes other populations, such as young children, non-Japanese speakers, or synesthetic individuals, would also improve understanding of the origin of sound symbolism. Although research on the neural mechanisms underlying sound symbolism is in its early stages, such research has the potential to advance our understanding of language.

Sound symbolism is not a marginal phenomenon in language. Developmental research has demonstrated that Japanese mothers often say mimetic words to their children [44], and sound symbolism of Japanese mimetic words promotes verb learning [18]–[20]. Sound symbolism may also play a key role in revealing the origin of language. Some researchers suggest that when human language started with our primitive ancestors, it began through their oral mimicking of the observed world [4], [17]. Thus, mimetic words may be similar to the words our ancestors used as a form of protolanguage. Sound symbolism, as a bridge between non-speech sound and conventional words, can provide new insights into the ontogenesis and phylogenesis of language.

## Supporting Information

Table S1 **Cortical activation for figure and motion (Experiment 2).**
(XLSX)
